# Condition transfer between prestressed bridges using structural state translation for structural health monitoring

**DOI:** 10.1007/s43503-023-00016-0

**Published:** 2023-08-02

**Authors:** Furkan Luleci, F. Necati Catbas

**Affiliations:** Department of Civil, Environmental, and Construction Engineering, University of Central Florida, Orlando, FL 32816 USA

**Keywords:** Structural state translation, Structural health monitoring, Domain generalization, Population-based structural health monitoring, Generative adversarial networks

## Abstract

Implementing Structural Health Monitoring (SHM) systems with extensive sensing layouts on all civil structures is obviously expensive and unfeasible. Thus, estimating the state (condition) of dissimilar civil structures based on the information collected from other structures is regarded as a useful and essential way. For this purpose, Structural State Translation (SST) has been recently proposed to predict the response data of civil structures based on the information acquired from a dissimilar structure. This study uses the SST methodology to translate the state of one bridge (*Bridge #1)* to a new state based on the knowledge acquired from a structurally dissimilar bridge (*Bridge #2*). Specifically, the Domain-Generalized Cycle-Generative (DGCG) model is trained in the Domain Generalization learning approach on two distinct data domains obtained from *Bridge #1*; the bridges have two different conditions: *State-H* and *State-D*. Then, the model is used to generalize and transfer the knowledge on *Bridge #1* to *Bridge #2*. In doing so, DGCG translates the state of *Bridge #2* to the state that the model has learned after being trained. In one scenario, *Bridge #2’s State-H* is translated to *State-D*; in another scenario, *Bridge #2’s State-D* is translated to *State-H*. The translated bridge states are then compared with the real ones via modal identifiers and mean magnitude-squared coherence (MMSC), showing that the translated states are remarkably similar to the real ones. For instance, the modes of the translated and real bridge states are similar, with the maximum frequency difference of 1.12% and the minimum correlation of 0.923 in Modal Assurance Criterion values, as well as the minimum of 0.947 in Average MMSC values. In conclusion, this study demonstrates that SST is a promising methodology for research with data scarcity and population-based structural health monitoring (PBSHM). In addition, a critical discussion about the methodology adopted in this study is also offered to address some related concerns.

## Introduction

Novel technologies and performance-based structural health monitoring (SHM) through innovative hybrid data interpretation frameworks have been explored to address risks related to safety and operation issues of civil structures (Catbas et al., 2022; Malekzadeh et al., 2015). Nevertheless, employing extensive monitoring systems on all civil structures with wide-range sensing layouts is an expensive and impractical approach, which would considerably limit the information flow from structures, making the data scarcity phenomenon a significant challenge in the SHM field (Luleci et al., 2022b). In addition, data transmission-related errors in SHM systems could occur during the monitoring process, causing loss of information (Zhuang et al., 2022). SHM applications' reliance on data-driven solutions makes the data scarcity phenomenon even more severe (Catbas and Malekzadeh 2016). Thus, estimating the health condition of one set of structure populations based on the SHM-related information obtained from a dissimilar set of structure populations has become an instrumental yet challenging goal for SHM (Von Haza-Radlitz et al., 2000).

Several studies have been conducted to address this issue in the Population-based SHM (PBSHM) subject (Catbas et al., 2002), which is aimed to increase the availability of physics- and data-based knowledge on one set of civil structures based on the information acquired from other structure populations. Nonetheless, the previous research had not adequately addressed the challenge with collecting information (e.g., signal response data) on different structural states of dissimilar structures until a recent exploration was made (Luleci & Catbas, 2022), which introduced a methodology named Structural State Translation (SST), purposed to obtain sensorial response data about unavailable states of dissimilar bridge structures based on the knowledge previously acquired from a different bridge structure.

This study would adopt the SST methodology to measure two dissimilar numeric prestressed concrete bridges, *Bridge #1* and *Bridge #2*, by translating the state (or condition) of *Bridge #2* to a new state based on the knowledge obtained from *Bridge #1*. In this sense, a Domain-Generalized Cycle-Generative (DGCG) model is trained on two distinct data domains, *State-H* (healthy) and *State-D* (damaged), acquired from *Bridge #1* in an unsupervised setting, with the cycle-consistent adversarial technique and the Domain Generalization (DG) learning approach implemented. Subsequently, the model is used to generalize and transfer its knowledge to *Bridge #2*. In this process, DGCG translates the condition of *Bridge #2* to the condition that the model learned after training; specifically, in one scenario, *Bridge #2’s State-H* is translated to *State-D*; and in another scenario, *Bridge #2’s State-D* is translated to *State-H*. Finally, the translated bridge states are evaluated by comparing them to the real states based on their modal parameters and Average MMSC (Mean Magnitude-Squared Coherence) values.

This paper would present the SST methodology in a more condensed, straightforward and simple format for readers, who are encouraged to consult the initial research literature (Luleci & Catbas, 2022) for more details about the methodology, such as SST's four major steps, i.e., preprocessing, training, translating and postprocessing, and the Domain Generalization learning method.

Many other learning approaches could be used for SST, such as Transfer Learning, Domain Adaptation, Zero-Shot Learning, and Meta-Learning. However, the Domain Generalization approach can provide more realistic and favorable results in real-world applications than the above approaches, as it does not leverage any information from the target domain in any form during the training process; but this also makes it more challenging than other learning approaches to deal with Out-Of-Distribution (OOD) domains (Blanchard et al., 2011; Muandet et al. 2013; Li et al., 2018; Zhou et al., 2021; Shen et al., 2021; Yang et al., 2021a). OOD is caused by semantic shifts (e.g., samples drawn from different classes) or covariate shifts (e.g., samples drawn from different data distributions). Since the modal parameters of *Bridge #1* and *Bridge #2* are different from each other (as shown in Fig. 4), it can be argued that the data domains collected from *Bridge #2* are OOD—the target domains, for which the DGCG model has to generalize its training knowledge. Section 5 would discuss the OOD domain generalization and the Degree of Dissimilarity (DoS) between civil structures.

The rest of the paper includes the following contents. First, the dataset used for the SST methodology is presented (Sect. 2). Then, the SST methodology is explained briefly, as introduced in the initial research (Sect. 3). Followingly, the accuracy of the translated states is evaluated (Sect. 4). Subsequently, a discussion of this study's results is presented (Sect. 5). Finally, a summary and conclusions are offered (Sect. 6).

## Dataset

The dataset used in the SST experiment is created from numeric bridge deck models as modeled and analyzed in the Finite Element Analysis (FEA) program. First, the bridge decks are modelled in the FEA program. Then, they are handled with Time History Analysis (THA) after Gaussian noise is applied. Subsequently, the acceleration response signals are extracted from the virtual sensor channels of each bridge deck model, so as to form the respective dataset for each bridge state, which will be employed in the SST methodology later. Finally, a modal identification process is performed using the datasets extracted from the models, in order to identify the physical meanings of each bridge deck model.

Model *Bridge #1* is adapted from NASA Causeway Bridge, a major connection between J. F. Kennedy Space Center and inland Florida, which consists of two separate bridges, Eastbound and Westbound. Each of them has a total length of 2993 ft and consists of 53 prestressed AASHTO Type II Girders spans at a length of 52 ft., with two flanking spans and a steel double-leaf bascule main span. The bridges are much structurally similar in terms of geometric and material properties and the topology (Debees et al., 2023; Dong et al., 2020; Luleci et al., 2022a). Thus, the structural parameters for the as-built condition of the bridges are expected to be the same. Model *Bridge #2*, on the other hand, is adapted from Bennett Causeway Bridge, which is located about 9 miles south of NASA Causeway Bridge. Both bridges go cross the same Indian River. Unlike NASA Causeway Bridge, Bennett Causeway Bridge is mostly modeled from Google Earth views based on engineering sense, as the structural plans of the bridge are not available. Regardless of the accuracy of the models and their ability of reflecting the actual structures, the main point of this study is to demonstrate the condition transfer between civil structures via SST.

**Fig. 1:**
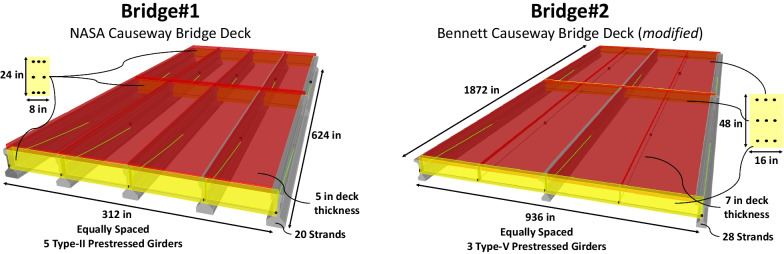
The Finite Element models of the bridge decks: *Bridge #1* is adapted from NASA Causeway Bridge, and *Bridge #2* is from Bennett Causeway Bridge

Having 6 AASHTO Type II girders in each bridge deck, Bennett Causeway Bridge is structurally similar to NASA Causeway Bridge, which has 5 AASHTO Type II girders. NASA Causeway Bridge is also composed of a steel double-leaf bascule in the middle. As both bridges are somewhat similar, some modifications are made to Model *Bridge #2*, so that it is more challenging for the SST methodology to demonstrate its potential further. For this purpose, the 6 AASHTO Type II girders are replaced with 3 AASHTO Type V girders in Model *Bridge #2*. Additional structural details of the bridge models can be seen in Fig. 1.

**Fig. 2:**
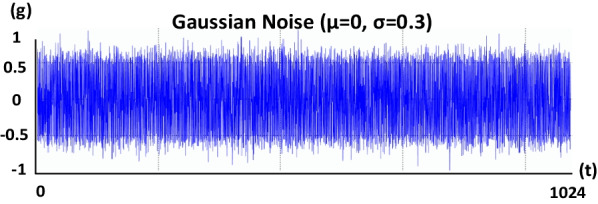
The excitation signal for the bridge deck models is applied in the form of Gaussian noise with the mean µ = 0 and standard deviation σ = 0.3

Four different bridge models are created: *State-H* and *State-D* of both *Bridge #1* and *Bridge #2*. *State-H* refers to the healthy condition, and *State-D* refers to the damaged condition of the bridges. The damage case is assumed to be 50% of the strands missing, in addition to 10% cross-section loss in the area of the middle girder, since in real-world conditions, the concrete spalling must occur before the corrosion reaches the strands. Then, to conduct the THA for each bridge model, a time history function is defined in the FEA program as an excitation signal to apply to each model. The signal is a Gaussian noise with its mean $\mu =0$ and standard deviation $\sigma =0.3$, lasting for 1,024 s (t), and it is sampled at a frequency (fs) of 256 Hz, as shown in Fig. 2. After applying the excitation signal to the bridge deck models, the THA for each model is carried out as per the defined history function.

**Fig. 3:**
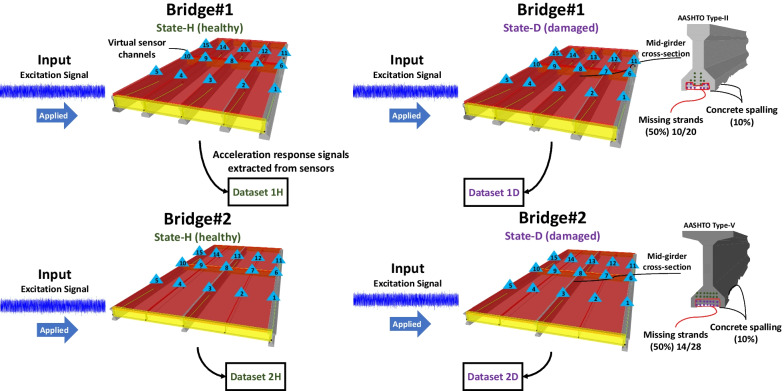
After the excitation signal is applied to each bridge deck model, THA is carried out for each model. Then, the response signals of the bridge decks are extracted, forming the respective datasets to be further employed in SST

Then, the acceleration response signals are collected from the virtual sensor channels of each model for the same (t) and (fs), so as to form the respective dataset for each state, as illustrated in Fig. 3. Each dataset consists of a 15-channel acceleration response signal. The datasets are denoted as *Dataset 1H*, *Dataset 1D*, *Dataset 2H*, and *Dataset 2D*, where the numbers (1 and 2) represent the bridge sequence and the letters (*H* and *D*) represent the state of the bridges, with *H* meaning "healthy" and *D* meaning "damaged".

**Fig. 4:**
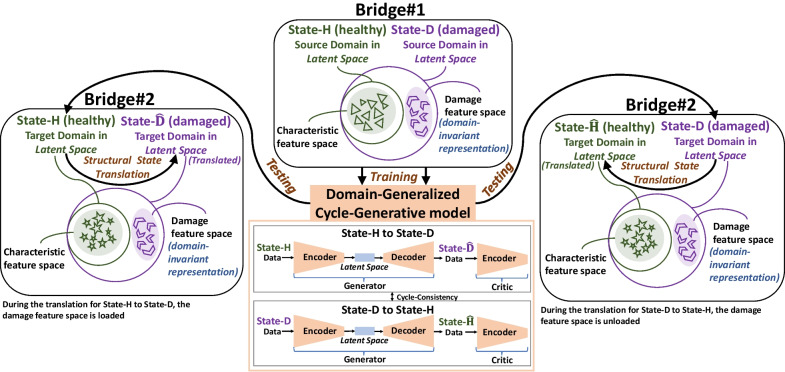
Structural State Translation applied to bridge decks: The DGCG model would learn the domain-invariant representation during training in the source domains of *Bridge #1* and then generalize knowledge to the target domains of *Bridge #2*, which is a dissimilar bridge. See Luleci and Catbas (2022) for more information about the training. The DGCG model used in this study is showcased in detail in Fig. 8. The DGCG model shown here is an abstract illustration just to indicate the location of the latent space in the model. Note that the model contains two networks, with one part responsible for translating *State-H* to *State-D* and the other part responsible for translating *State-D* to *State-H*.

Each dataset defines a data domain; e.g., the dataset collected from *State-H* of *Bridge #1* (*Dataset 1H*) defines one data domain, and the dataset of *State-D* of *Bridge #1* (*Dataset 1D*) defines another. The similar process goes for all other datasets. As illustrated in a representative figure, Fig. 4**,** the circle diagrams whose boundaries are defined by the symbolic shapes can be considered as the source and target domains, representing latent/embedding space of the datasets obtained from the bridge deck states. It should be noted that the source domains are the domains on which the DGCG model is trained, and the target domains are the domains to which the model generalizes knowledge after training. These shapes in the domains showcase the changes with the characteristic properties of the response data collected from different bridges, since the “Degree of Similarity” (DoS) between the bridge decks would affect the statistical and/or dynamic characteristics of the response datasets (e.g., different mode shapes, etc.).

The invariant representations in the data domains have not become well-established research subjects yet, nor an active research area in AI. And most invariant-based learning approaches have been put up based on intuition. Section 5 will discuss the domain-invariant representations.

The DGCG model is then trained on the source domains and learns this invariant space before it generalizes knowledge to the target domains by translating the structures’ states, specifically their data domains. During the translation, the model would “enforce” the damage feature space learned during the training. The enforcing action can be considered as “loading” or “unloading” the damage feature space in the respective domains throughout the encoding and decoding process in the generator. For example, when shifting *State-H* to *State-D*, the damage feature space would be loaded; when shifting *State-D* to *State-H*, the damage feature space would be unloaded.

Accordingly, while the triangular- and star-like shapes define the characteristic feature spaces of *Bridge #1* and *Bridge #2*, which are intrinsic to the data domains of the healthy bridges (*State-H*), the chevron-like shapes would define the damage feature spaces, which are intrinsic to the data domains of the damaged bridge (*State-D*). For damage feature spaces, the damage refers to 50% of the strands missing, in addition to 10% cross-section loss in the area of the middle girder.

Readers can consult research by (Shen et al., 2021; Yang et al., 2021a; Ye et al., 2021; Zhou et al., 2021) for more information about DoS, domain-invariancy, and OOD generalization. Section 5 in this paper would briefly discuss these subjects; a complete exploration can be found in research by (Luleci & Catbas, 2022).

Furthermore, understanding the physical representations of the bridges is important in the SHM context. Hence, a modal identification process is performed in the Artemis^®^ for the datasets extracted from each bridge model, the parameters of which are identified by using the *Stochastic* Subspace Identification technique (van Overschee and de Moor, 1993) with an Extended Unweighted Principal Component (SSI-UPCX). Then, additional covariance information is used to make a better prediction of the final set of modes than the typically averaged stable modes of different model orders, so as to get the final prediction. The SSI-UPCX method is adopted, with 66% Hann window overlapping and a resolution of 4096 frequency lines for the dataset of each bridge deck model.

**Fig. 5:**
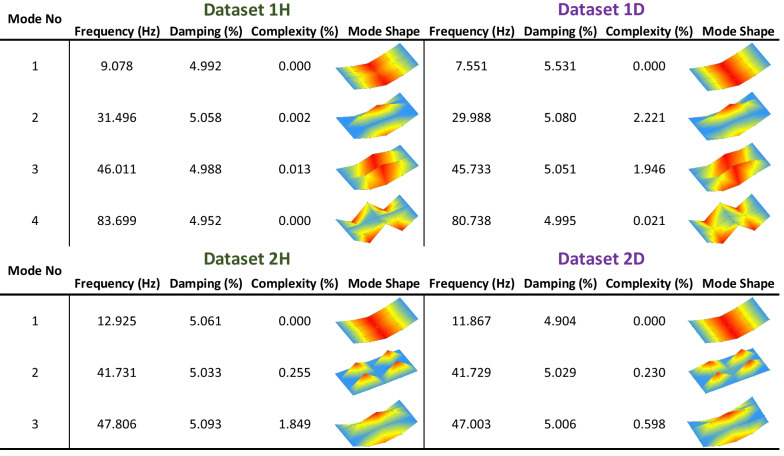
The modal parameters of *State-H* and *State-D* of *Bridge #1* and *Bridge #2* after implementation of SSI-UPCX

The modal parameters of *State-H* and *State-D* of *Bridge #1* and *Bridge #2* obtained in the SSI-UPCX method are given in Fig. 5. A total of four modes are identified for *Dataset 1H* and *Dataset 1D* (i.e., the datasets of *Bridge #1*); on the other hand, a total of three modes are obtained for *Dataset 2H* and *Dataset 2D* (i.e., the datasets of *Bridge #2*). No clear modes are observed after Mode 3 in the datasets of *Bridge #2*. As seen in Fig. 5, the modal parameters of *Bridge #1* and *Bridge #2* are significantly different, and so are Modes 2–4; therefore, it can be reasonably assumed that the bridge deck structures are dissimilar. It is also observed that the Complexity (%) values are low, indicating that the mode shapes' minimum and maximum values would appear simultaneously. Making the minimal Complexity (%) of the mode shapes is preferable. The mode shapes may be complex due to inconsistent data/bad measurements, poor modal estimation, or non-proportional damping. Complexity is defined by the Modal Complexity Factor (MCF), as given in Eq. (1).

1 $$\mathrm{MCF}=1-\frac{{\left({S}_{xx}-{S}_{yy}\right)}^{2}+4{S}_{xy}^{2}}{{\left({S}_{xx}+{S}_{yy}\right)}^{2}},$$

where ${S}_{xx}=Re{\{\psi\}}^{T}Re\{\psi\}$, ${S}_{yy}=\mathrm{Img}{\{\psi\}}^{T}\mathrm{Img}\{\psi\}$, and ${S}_{xy}=Re{\{\psi\}}^{T}\mathrm{Img}\{\psi\}$; and $Re$ indicates the real data, $Img$ indicates the imaginary data, and $\{\psi\}$ indicates the mode vector. The closer to 0 the MCF value, the more real the mode; and the closer to 1 the MCF value, the more complex the mode. The minimum and maximum mode values appear simultaneously (in-phase or out-of-phase) when the modes are real; e.g., the degree of freedom of the modes would move at the same time in the same direction (in-phase) or at the same time but in opposite directions (out-of-phase). However, when the modes are complex, the mode values would occur between in-phase and out-of-phase.

## Structural state translation

**Fig. 6:**
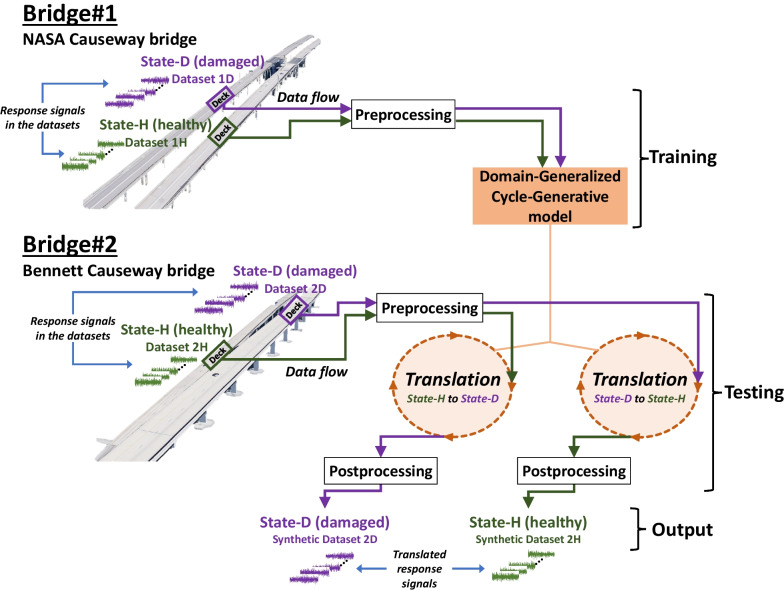
SST is implemented on the two bridge decks with different bridge structures on Florida's Atlantic Coast. First, the acceleration response datasets of the healthy and damaged bridge decks of NASA Causeway Bridge are used in preprocessing. Then, the datasets are fed into the DGCG model (containing two networks: one for translating *State-H* to *State-D* and other for translating *State-D* to *State-H*) for training under an unsupervised setting in the cycle-consistent adversarial approach. Later, the response datasets collected from the healthy deck of Bennett Causeway Bridge are preprocessed, used in the translation phase, and then again utilized for the postprocessing phase. Ultimately, the healthy bridge deck is translated to the damaged bridge deck (*State-H* to *State-D*). The same SST procedure is also carried out for the damaged deck of Bennett Causeway Bridge, so as to translate the damaged bridge deck to the healthy bridge deck (*State-D* to *State-H*).

This study is aimed at demonstrating that condition transfers between bridges via SST are viable. The SST methodology implemented in this study is conceptually illustrated in Fig. 6. As mentioned earlier, the components of NASA Causeway Bridge are structurally identical in geometric and material properties and topologies. Thus, the structural parameters of the as-built conditions of the bridge components are the same (Dong et al., 2020; Luleci et al., 2022a), allowing the use of two distinct data domains (e.g., undamaged and damaged) from different bridges in the DGCG model. The fact that the bridge decks are the same and only the data related to the structural conditions are different would facilitate the model to learn specifically the data domain-wise differences (the structural condition—damage feature space) after trained with the data domains of both *State-H* and *State-D* of *Bridge #1*.

**Fig. 7:**
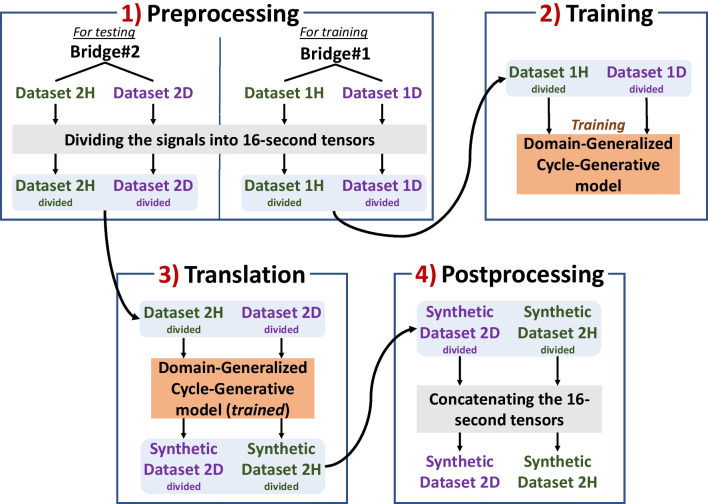
The SST methodology consists of four steps: preprocessing, training, translation, and postprocessing

As mentioned in the Introduction section, readers are encouraged to consult the initial research for more details about the SST methodology as well as OOD generalization. Accordingly, this paper discusses SST in a condensed, straightforward and simple format. The framework of SST consists of four steps: (1) Preprocessing, (2) Training, (3) Translation, and (4) Postprocessing, as visualized in Fig. 7.

### Preprocessing

In the Preprocessing step, the 1,024-s response signal for each sensor channel in the datasets (*Dataset 1H*, *Dataset 1D*, *Dataset 2H,* and *Dataset 2D*) is divided into 16-s tensors, resulting in 64 times the 16-s tensors per sensor channel. Implemented for a more efficient training procedure, this approach has also been used in several other studies (Avci et al., 2017; Luleci et al., 2021, 2023a, 2023b; Luleci & Catbas, 2022). After dividing the signal into tensors, the datasets, which are now consisting of 16-s tensors per sensor channel, are named *Dataset divided 1H*, *Dataset divided 1D*, *Dataset divided 2H* and *Dataset divided 2D*.

### Training

**Fig. 8:**
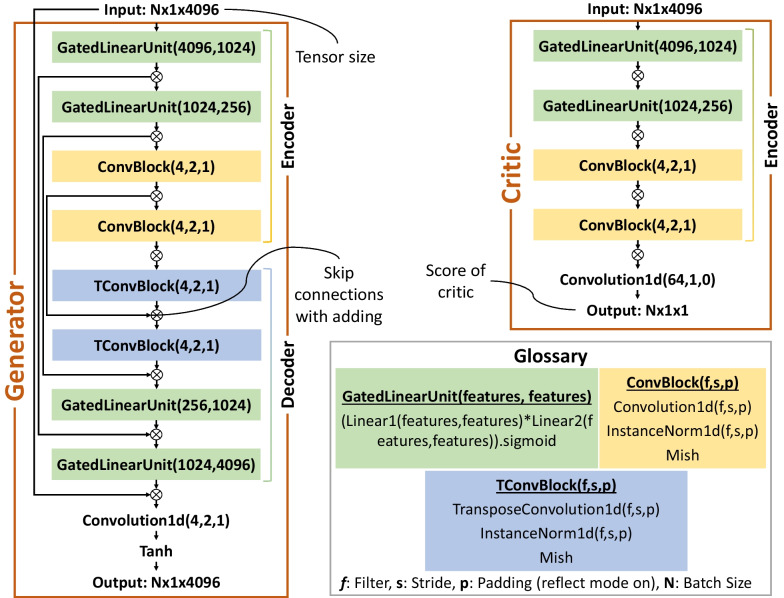
The single DGCG network architecture of the Domain-Generalized Cycle-Generative (DGCG) model. This network is the same for both processes of translating *State-H* to *State-D* and translating *State-D* to *State-H*, delivering two networks in total (DGCG model)

In the Training step, the DGCG model is trained on *Dataset divided 1H* and *Dataset divided 1D*. In other words, the model is trained with 960 tensors from *State-H* of *Bridge #1* and 960 tensors from *State-D* of *Bridge #1*. Here, the number of 960 comes from the multiplication of 64 times the 16-s tensors with a total of 15 sensor channels. The DGCG model used in this study, however, is improved to perform more efficiently than the models proposed in the initial research. As such, the number of learnable model parameters is reduced from 80 million to 53.7 million (with fewer parameters, the training time will be short). Separating the mapping networks and positioning them both in the encoders and decoders would help accomplish this efficiency, compared to only using them in the encoders (Luleci & Catbas, 2022). Moreover, the residual blocks are removed from the latent space, so as to improve the model’s learning process in terms of training time with the same training performance. The single DGCG network architecture is shown in Fig. 8, which has two same networks, due to the cycle-consistent adversarial training nature. For instance, one network is responsible for translating *State-H* to *State-D,* and the other is for translating *State-D* to *State-H*. The model hyperparameters used in this study are presented in Table 1.

**Table 1:** Parameters of the domain-generalized cycle-generative (DGCG) model

Model parameter	Description
Batch size (N)	4
Epoch	200
Learning rate (for both networks)	2 × 10^–4^
Critic Iteration per Epoch	5
${\lambda }_{Id}$ (identity loss)	10
${\lambda }_{Cyc}$ (cycle loss)	10
${\lambda }_{GP}$ (gradient penalty)	10
Optimizer	AdamW

The model is trained in an unsupervised setting by using the cycle-consistent adversarial technique (Zhu et al., 2017), which is the same as in the initial research (Luleci & Catbas, 2022). Specifically, the model is iteratively trained on the two datasets (*Dataset 1H divided*, and *Dataset 1D divided*) to decrease discrepancies in representation between the data domains; in particular, the feature space is to be domain-invariant across different domains. This makes the learned model generalizable and enables it to transfer its knowledge to other unseen target domains. It is also important to note that the model is trained without leveraging any information from the target domain in any way (e.g., there is no fine-tuning of the model based on the test data), as Domain Generalization requires learning without access to the test data (Lu et al., 2022). The loss functions and the training data workflow can be found in the initial research. In summary, the only portion that has been changed in the SST methodology in this study is the DGCG model, which is made more efficient, as explained above.

To monitor the learning performance of the model during the training of DGCG, some indices are used, including Fréchet Inception Distance (FID) (Heusel et al., 2017) and Mean Magnitude-Squared Coherence (MMSC), along with a total generator and critic losses. The details about the formulation of the indices and how they are used can be found in the initial research. At the end of the training process, as the generator and critic losses and FID values are approaching 0, the MMSC value is stabilized around 0.99, which indicates that the translated 16-s tensors are almost identical to the original 16-s tensors, because the MMSC of 1 suggests a complete similarity between the tensor pairs. Therefore, it can be concluded from the training results that the DGCG model has learned one-to-one mapping between the healthy and damaged domains (*State-H* and *State-D*).

**Fig. 9:**
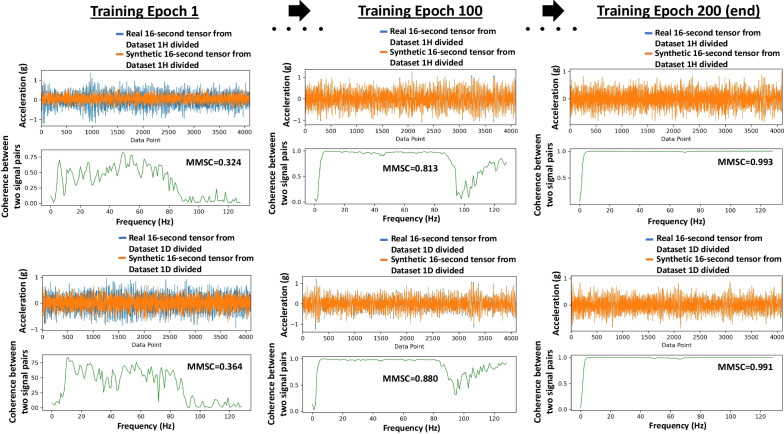
The plots of the real and translated (synthetic) 16-s tensors in the time domain and their coherences for both *Dataset 1H divided* and *Dataset 1D divided* at Training Epochs 1,100 and 200 (the end of the training). MMSC is the mean of the segments of all the coherence estimates of the signal pairs. When MMSC equals 1, it indicates a total similarity; and 0 indicates dissimilarity

Figure 9 illustrates the 16-s tensors from *Dataset 1H divided,* and *Dataset 1D divided,* as shown during the training for Epoch 1, Epoch 100, and Epoch 200 (the training is ended at Epoch 200). The time domain in Fig. 9 shows the original tensors from *Dataset 1H divided* and *Dataset 1D divided,* as well as the translated versions of those tensors (denoted as synthetic) during the training. In addition, the coherence of the translated and the original tensors is measured and plotted, respectively in the figure, with the MMSC values in each coherence plot also presented. MMSC simply takes the mean of all the segments of the coherence estimates of the 16-s tensor pairs. As revealed in Fig. 9, after training, the DGCG model can translate the domains of 16-s tensors from *State-D* to *State-H* and *State-H* to *State-D*, since MMSC gets close to 1; and the signals look alike in the time domain. However, this is true only for *Bridge #1* for now, as it is unknown how the model will perform for the datasets of *Bridge #2*. MMSC is a great index for quick similarity checks of signal pairs, and this is why it is used in training. The translated bridge states (*Bridge #2*) are evaluated by comparing their modal parameters to the ground truths, as shown in Sect. 4.

### Translating

After DGCG is trained, a typical domain-translation procedure (de Bézenac et al., 2019; Hu et al., 2021; Yang et al. 2021b) is implemented in the Translation phase, where the 16-s tensors in *Dataset 2H divided* and *Dataset 2D divided* are fed into the trained DGCG for domain-translation, as shown in the Translation part in Fig. 7. As such, the domain of the tensors is translated from *State-H* to *State-D* and also from *State-D* to *State-H*. Then, the domain-translated 16-s tensors in each sensor channel are concatenated together, as explained in the next section.

### Postprocessing

In the Postprocessing phase, a reverse process of Preprocessing is implemented. The translated (or generated) 16-s tensors in each sensor channel in *Synthetic Dataset 2D divided* and *Synthetic Dataset 2H divided* are concatenated back to re-form the 1,024-s signals. As a result, the final form of the datasets of *Synthetic Dataset 2H* and *Synthetic Dataset 2D* now consists of domain-translated 1,024-s response signals, as shown in the Postprocessing part in Fig. 7.

**Side note** It is noticed in this study that concatenating the 16-s tensors in a shuffle mode (during the test) would result in very different modal identifiers. In other words, if feeding the 16-s tensors from each sensor channel in the “trained” DGCG model to translate the domain of those tensors and then concatenating the translated 16-s tensors randomly to re-form the 1,024-s signals, the characteristics of the signals would be disrupted, leading to different modal parameters. Thus, the concatenation of the 16-s tensors has to be made in order, e.g., firstly, translating the first 16-s tensor of a 1,024-s tensor; secondly, translating the second one; thirdly, waiting for the sixty-fourth being delivered; finally, concatenating them in order, so as to form the full 1,024-s signal. However, performing the training procedure in a shuffle mode is essential, as it can increase the model's generalization ability.

## Evaluation of the results of structural state translation

**Fig. 10:**
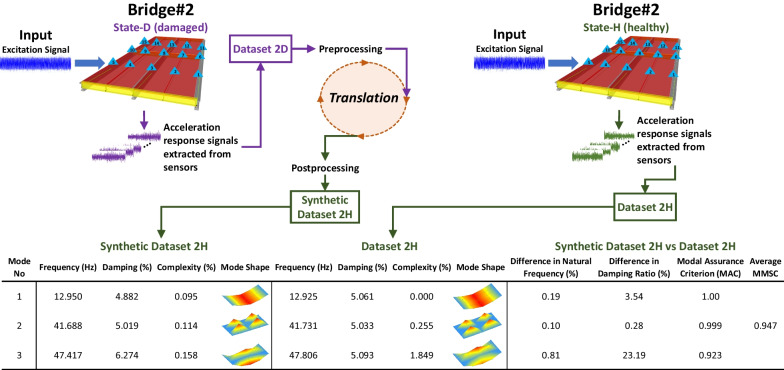
The modal parameters of *Synthetic Dataset 2H* and their comparison with those of *Dataset 2H*

**Fig. 11:**
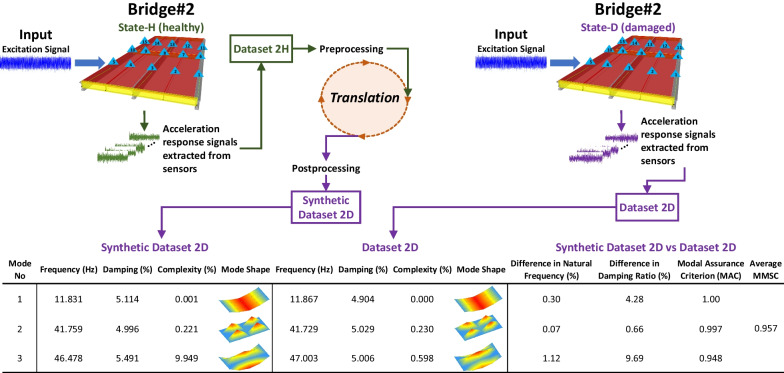
The modal parameters of *Synthetic Dataset 2D* and their comparison comparing them with those of *Dataset 2D*

The modal parameters of the translated states of *Bridge #2* (*Synthetic Dataset 2D* and *Synthetic Dataset 2H*) are compared with those of the original states of *Bridge #2* (*Dataset 2D* and *Dataset 2H*). The real modal parameters of the bridges are also given in Fig. 5. The modal identification procedure for the synthetic datasets is implemented in the same way as for the real datasets (Sect. 2). Figures 10, 11 present the modal parameters and mode shapes of *Synthetic Dataset 2H* and *Synthetic Dataset 2D,* as well as their percentage differences in the modal parameters of the real datasets (*Dataset 2H* and *Dataset 2D*). Specifically, the Difference in Natural Frequency (%), Difference in Damping Ratio (%), and Modal Assurance Criterion (MAC) are presented in the figures. The Average MMSC values are also provided, which take the average of all the MMSC values of the 1,024-s signal pairs from synthetic and real datasets. The MMSC is also found really useful and intuitive in Luleci & Catbas (2022) during and after the training process, as it shows the average similarity between the translated signals and the original ones.

Figures 10 and 11 demonstrate that the modal parameters of the translated bridge states (*Synthetic Dataset 2H* and *Synthetic Dataset 2D*) are significantly similar to those of the real bridge states (*Dataset 2H* and *Dataset 2D*). As such, the modes of the translated and real bridge states are similar, up to 0.07% in natural frequencies, 0.28% in damping ratios, 1.00 in MAC (Modal Assurance Criterion) values, and 0.957 in Average MMSC values. On the other hand, the largest difference in the natural frequency is 1.12%, the largest difference in damping ratio is 23.19%, the lowest MAC is 0.923, and the lowest Average MMSC is 0.957; and lastly, the complexity values are observed to be very minimal, with the highest value of 9.949%, making the modal identification results reliable. Overall, it can be concluded that the modal parameters obtained from the synthetic datasets deliver significant similarity to those obtained from the real datasets, with some minimal variations.

Using the SST methodology entails an acceptable error margin, because there will be no information about the ground truth in real-world scenarios; e.g., if we have the *Synthetic Dataset 2D,* for evaluation, we can compare it with the real dataset, *Dataset 2D*; however, we will not have the real dataset in real life. It is known that when damage exists in a structure, its natural frequencies would decrease, as observed in Fig. 5 for the modal parameters of the real datasets. Although the natural frequencies of the synthetic datasets are very close to those of the real datasets, the frequency of Mode 2 in *Synthetic Dataset 2D* (41.759 Hz) should be lower than that of Mode 2 in *Synthetic Dataset 2H* (41.688 Hz), as *Synthetic Dataset 2D* is the damaged case and *Synthetic Dataset 2H* is the healthy case. Therefore, establishing an acceptable error margin is essential for effectively implementing SST.

As to the methodology itself, it should also be pointed out that the DGCG model comprises two networks: one network (*N*_*1*_) is responsible for translating *State-H* to *State-D*, and the other (*N*_*2*_) for translating *State-D* to *State-H*, as mentioned in Sect. 3. Arguably, when the data from an unknown state are fed to *N*_*1*_, it will output data for *State-D*, regardless of the input state's identity; similarly, if the data from the unknown state are fed to *N*_*2*_, it will yield data for *State-H*. In this regard, knowing the state of a structure, whether it is damaged or not, would not matter for the model. Inputting several unknown states and interpreting them fall in the scope of future studies.

## Discussion: SST, DG, OOD, and DoS

The SST methodology uses domain-adversarial learning to align the source domains. The learner, DGCG, minimizes discrepancies among the source domains to learn domain-invariant representations; and then, it generalizes knowledge on the target domains under the covariate shift (Out-Of-Distribution, i.e., OOD). It should be noted that since DGCG is trained in an unsupervised setting in this study (with no labels), the covariate shift is the main reason for OOD.

The existing research (Ben-David et al., 2007; Blanchard et al., 2011; Muandet et al. 2013; Deshmukh et al., 2019; Albuquerque et al., 2019) has theoretically proven that the feature representations invariant to source domains are general and transferable to other related target domains. This finding raises a question: Would a representation learned to be invariant to the source domain shift be guaranteed to be able to generalize knowledge to any shifts in any target domains? Another concern is the degree of relatedness of the target domains to the source domain for good generalization.

While it is intuitive to learn the domain-invariant representations in the source domains and generalize them to the target domains that are OOD, how to identify the existence, degrees, types, and learnability of invariant representations across domains that can guarantee OOD generalization remains underexplored as an early research area (Shen et al., 2021; Yang et al., 2021a; Zhou et al., 2021). In this regard, defining the theoretical characterization of the learnability of a problem is a simple goal in the ML field, and most of the efforts have been made in the *i.i.d.* setting. However, the existing literature has not shown how to define such theoretical characterization of the learnability of a problem to generalize knowledge to OOD domains. Identifying the learnability of the OOD domains is extremely difficult, since it is almost impossible to enable learners to generalize knowledge for arbitrary and unknown distributions. Therefore, it is very important to develop new theories to reveal how minimizing the discrepancies in the source domain can improve the generalization in the OOD domains.

This study demonstrates that it is possible to conduct SST between dissimilar (“to some degree”) civil structures. Nonetheless, as there is no sufficient literature to offer firm theoretical understanding of generalization to the OOD domains, this study assumes the existence of learnable domain-invariant representations in the acceleration response datasets of *Bridge #1*, so that learners can generalize related knowledge to the target domains that are OOD to some extent. Conversely, such domain-invariant representations may not exist, even if they are learnable. Questions that are underexplored in the existing literature include: when can learners learn and when can they not learn? If learners have learned them, under what degree and in what kinds of distributional shifts in the target domain can learners make good generalizations?

The Degree of Similarity (DoS) between the bridge structures discussed in this study is one component that remains in a grey zone. Another issue is how this DoS would influence the distributional characteristics of the acceleration response signals collected from these structures. This study presumes that the bridge structures are dissimilar based on the differences between their modal parameters. Figure 4 shows that the modes of *Bridge #1* and *Bridge #2* are quite different. Consequently, under this presumption, the response datasets collected from different states of different bridge decks, each of which is a data domain, are OOD to each other. On this basis, if the modal parameters of two acceleration response datasets are different, the structures will also be different, which is caused by the distributional differences of the response signals in those datasets. Nevertheless, how to achieve DoS in a quantitative space between those bridges remains a big question, which has been well explored by the existing research (2021b; Gosliga et al., 2021a; Wickramarachchi et al., 2022).

Although this study offers a successful SST methodology for dissimilar bridges, the theoretical understanding of OOD generalization problems is limited and primarily applied on an intuitive basis. For example, while it is intuitive to think that domain invariance should exist between two prestressed bridges that are generally similar but still with certain dissimilarities, it is hard to grasp the idea of implementing SST or any other data-driven knowledge transfer techniques between different types of civil structures, e.g., between a prestressed concrete-to-steel truss bridge and a steel tower-to-residential timber structure.

While DoS between civil structures is related to the OOD generalization, the existing literature's understanding of this “degree” is limited. Thus, the exploration in the Population-based SHM research area proves significantly valuable.

In view of the discussion above, an overarching question remains to be answered: What should be the degree of similarity between civil structures, so that SST or other knowledge-transfer techniques between structures could be implemented?

A more extensive survey into the abovementioned subjects is presented in a research by (Luleci & Catbas, 2022).

Lastly, the real-world structures may have numerous possible states; e.g., there are varying levels of damage, coupled with different damage classes at different locations, as well as changing structural states due to varying environmental factors. The model used in this study is trained with the response datasets of only two states, so it might not successfully translate the data domains for different states of different structures. Training the AI model with relevant information is needed when the objective is to translate data domains of structures to different states, such as various section losses, boundary conditions, or conditions caused by other external stressors. In real life, collecting such an amount of data to train models is not feasible. One solution might be building a numerical model (calibrated) of a particular structure to generate acceleration response datasets for the desired structural states (just as in this study). Then, these datasets can be used to train models, in order to obtain a model that can perform SST for the desired states of different structures.

## Summarization, conclusion and final remarks

In order to make better and objective decisions on safety, maintenance and operation, civil structures need to be monitored, tracked and assessed. Various studies have demonstrated that employing SHM systems on civil structures is very valuable in tracking their conditions. However, data collection procedures with extensive sensing layouts may be expensive and impractical, leading to the data scarcity phenomenon in the SHM field. This phenomenon would become more critical as SHM is implemented in data-driven techniques. To tackle this challenge, Population-Based SHM (PBSHM) has been introduced to boost the data availability within populations of structures.

Nevertheless, the existing research had not sufficiently dealt with the challenge of accessing the information on different structural states of dissimilar structures until a recent exploration was implemented by Luleci & Catbas (2022), who proposed Structural State Translation (SST), aimed to obtain the sensorial response data for unavailable states of dissimilar bridge structures based on the knowledge acquired from a different bridge structure. Readers are advised to consult the initial research results for more details about the methodology. Accordingly, this paper presents the SST methodology from different case studies in a more condensed, straightforward, and simple format.

This study applies SST on two dissimilar numeric prestressed concrete bridge deck models, *Bridge #1* and *Bridge #2*, with a purpose to translate the state of *Bridge #2* to a new state based on the knowledge obtained from *Bridge #1*. In this regard, DGCG is trained on two different data domains acquired from *Bridge #1*, i.e., *State-H* and *State-D*, under an unsupervised setting in a cycle-consistent adversarial technique, which takes the Domain Generalization learning approach. Then, DGCG is used to generalize and transfer its knowledge to *Bridge #2*. In doing so, DGCG translates the state of *Bridge #2* to the state that the model has learned in training.

An evaluation of the translated states is carried out by comparing the modal parameters of *Bridge #2*’s translated states to the real states. The comparison results demonstrate that the translated bridge deck states are significantly similar to the real states, with a max difference of 1.12% in their natural frequencies, a difference of 0.28% in their damping ratios, a minimum MAC of 0.923, and an Average MMSC value of 0.947.

Although the results of this study are well founded, there are additional considerations to be further investigated, as addressed in Sect. 5 in detail for the OOD generalization in SST of civil structures. It is intuitive to assume that the domain invariancy should exist between the two prestressed bridges, which are similar to a degree. Yet, it is difficult to grasp the notion of using SST or any other data-driven knowledge transfer techniques between different civil structures, such as between prestressed concrete and steel truss bridges or between steel towers and residential timber structures. While the Degree of Similarity (DoS) between civil structures is relates to the OOD generalization, the understanding of this “degree” has been poorly addressed in the existing literature. Thus, explorations in the PBSHM research field hold significant importance.

## Data Availability

Some or all used models, codes, and detailed results are available from the authors of this paper upon request.

## Glossary

State: State and condition are interchangeably used to refer to the structure's condition.
Domain: A domain is a group of data that are held by an attribute. The acceleration response datasets collected from each bridge state defines a data domain, e.g., the dataset collected from *State-H* of *Bridge#1* defines one data domain, and the dataset from *State-D* of *Bridge#1* defines another.
Source Domain: The source domain refers to the domain used for the training.
Target Domain: The target domain refers to the domain used for the test.
Translation: Translation refers to the data domain translation process, e.g., when translating *Bridge#2*’s *State-H* to *State-D*, their data domains are being translated.
*State-H*: *State-H* is the healthy condition of the bridge.
*State-D*: *State-D* is the damaged condition of the bridge, where the damage case is assumed to be 50% of the strands missing in addition to 10% cross-section loss in the area of the middle girder.
*State-*$\widehat{H}$: *State-*$\widehat{H}$ is the translated version of *State-H*, whose data domain is translated from *State-D*.
*State-*$\widehat{D}$: *State-*$\widehat{D}$ is the translated version of *State-D*, whose data domain is translated from *State-H*.
Out Of Distribution: When a classifier is tested on a target domain whose labels of the data samples (semantic) and/or data distributions (covariate) are different from the source domain, then the target domain is Out-Of-Distribution (OOD).
Unseen Domain: An unseen domain is the data domain that the classifier is not trained with. Some learning approaches, e.g., Transfer Learning, leverage knowledge from the target domain during training. Yet, this is not the case for the unseen domain.
Degree Of Similarity: The Degree of Similarity (DoS) is the relatedness of a structure pair in a quantifiable space regarding the structure’s overall geometry, material, and topology.
